# Clinical course of neurogenic bladder dysfunction in human T-cell leukemia virus type-1-associated myelopathy/tropical spastic paraparesis: a nationwide registry study in Japan

**DOI:** 10.1186/s13023-021-01990-3

**Published:** 2021-08-09

**Authors:** Naoki Iijima, Junji Yamauchi, Naoko Yagishita, Natsumi Araya, Satoko Aratani, Kenichiro Tanabe, Tomoo Sato, Ayako Takata, Yoshihisa Yamano

**Affiliations:** 1 Division of Neurology, Department of Internal Medicine, St. Marianna University School of Medicine, Kawasaki, 2168511 Japan; 2 Department of Rare Diseases Research, Institute of Medical Science, St. Marianna University School of Medicine, Kawasaki, Japan; 3 LSI Medience Co., Tokyo, Japan; 4 Department of Preventive Medicine, St. Marianna University School of Medicine, Kawasaki, Japan

**Keywords:** Human T-cell leukemia virus type 1, Human T-cell leukemia virus type 1-associated myelopathy/tropical spastic paraparesis, Neurogenic bladder, Urinary symptom score, Mirabegron

## Abstract

**Background:**

Most patients with human T-cell leukemia virus type 1-associated myelopathy/tropical spastic paraparesis (HAM/TSP) develop neurogenic bladder dysfunction. However, longitudinal changes and treatment effects remain poorly understood. This study aimed to characterize the clinical course of urinary dysfunction in this population.

**Methods:**

This prospective observational study included 547 patients enrolled in HAM-net, a nationwide registry for HAM/TSP in Japan. Urinary dysfunction severity was evaluated using the HAM/TSP-bladder dysfunction symptom score (HAM-BDSS) and the HAM/TSP-bladder dysfunction severity grade (HAM-BDSG). These specific measures were recently developed for assessing urinary dysfunction in HAM/TSP. We analyzed longitudinal changes over a 6-year follow-up period, associations between urinary and gait dysfunction, and treatment efficacy of urinary catheterization and mirabegron (a β3-adrenergic agonist for overactive bladder symptoms).

**Results:**

The mean (standard deviation [SD]) age and disease duration at enrollment were 61.9 (10.7) years and 16.6 (11.6) years, respectively, and 74.6% of patients were women. Only 8.0% were free from urinary symptoms (HAM-BDSG 0), 65.4% had urinary symptoms or were on medication (HAM-BDSG I), and 23.2% and 3.3% used intermittent and indwelling catheters (HAM-BDSG II and III), respectively. HAM-BDSG and BDSS were worse in patients with greater gait dysfunction (*p* < 0.001 for both). During the 6-year follow-up, 66.7% of patients with HAM-BDSG 0 developed new urinary symptoms. Of those with HAM-BDSG I at enrollment, 10.8% started using urinary catheters. Importantly, HAM-BDSS significantly improved after initiating catheterization (mean [SD] change, − 8.93 [10.78], *p* < 0.001). The number of patients receiving mirabegron increased in the fourth year. Multivariable linear regression analysis significantly associated mirabegron with improvement in HAM-BDSS (− 5.82, 95% confidence interval − 9.13 to − 2.51, *p* = 0.001).

**Conclusions:**

Urinary dysfunction affected 92% of patients and progressed over the 6-year follow-up. Urinary symptoms were more severe in patients with poorer gait function. Urinary catheterization and mirabegron were effective in relieving symptoms. Effective utilization of real-world data is key to establishing evidence for rare diseases, such as HAM/TSP.

**Supplementary Information:**

The online version contains supplementary material available at 10.1186/s13023-021-01990-3.

## Background

Human T-cell leukemia virus type 1 (HTLV-1)-associated myelopathy/tropical spastic paraparesis (HAM/TSP) is a rare neurological disease. It develops in a small proportion of people infected with HTLV-1 [1–3]. There are no radical treatments for HAM/TSP. Thus, symptom management is the only treatment option for patients [4]. Because HAM/TSP causes chronic inflammation, which destroys the spinal cord, particularly at the thoracic level, the main symptoms are progressive gait disturbance and neurogenic bladder dysfunction [3]. Most patients with HAM/TSP develop urinary symptoms, including storage and voiding symptoms [5–11]; moreover, some patients require urinary catheterization [9, 12]. Therefore, urinary dysfunction severely impacts the daily lives of patients, and establishing an effective treatment is imperative.

Longitudinal and quantitative clinical data are necessary to determine the clinical characteristics and establish better disease management. However, there is limited information regarding neurogenic bladder in this patient population because of the rarity of HAM/TSP and the lack of standardized assessment tools. Most previous reports have been cross-sectional studies with relatively small sample sizes. Moreover, although various urinary symptom measurements exist, such as the International Prostate Symptom Score (I-PSS) and the Overactive Bladder Symptom Score (OABSS) [13, 14], comprehensive evaluation of neurogenic bladder dysfunction is difficult because of symptom heterogeneity. Symptom scores for neurogenic bladder have been reported. For example, the neurogenic bladder symptom score (NBSS) has been developed for patients with multiple sclerosis, spinal cord injury, and spina bifida [15–17]. In addition, assessment tools for urinary dysfunction in patients with HAM/TSP, namely, the HAM/TSP-bladder dysfunction symptom score (HAM-BDSS, Additional file 1: Table S1) and HAM/TSP-bladder dysfunction severity grade (HAM-BDSG, Additional file 1: Figure S1), have also recently been developed [18]. However, there are no reports using these measures to evaluate the clinical course of urinary dysfunction in patients with HAM/TSP.

This study aimed to characterize the clinical course of neurogenic bladder dysfunction in patients with HAM/TSP. We used the HAM-BDSS and HAM-BDSG to analyze urinary dysfunction in more than 500 patients enrolled in HAM-net, a nationwide registry for patients with HAM/TSP in Japan [19]. We also assessed the efficacy of urinary catheterization and mirabegron (a newer class of medication for overactive bladder symptoms [20]) to relieve urinary symptoms.

## Methods

### Data source and study population

Data from HAM-net were analyzed (UMIN000028400) [19]. The Additional file 1: Methods section presents details of HAM-net. Briefly, HAM-net prospectively collects patient data, including demographic information and medical conditions, such as symptoms, comorbidities, and medications, through annual telephone interviews with patients conducted by trained nurses and coordinators. Owing to the fact that HAM-net does not have any recommendations or protocols for examination and treatment, patients are treated at the discretion of doctors.

**Fig. 1:**
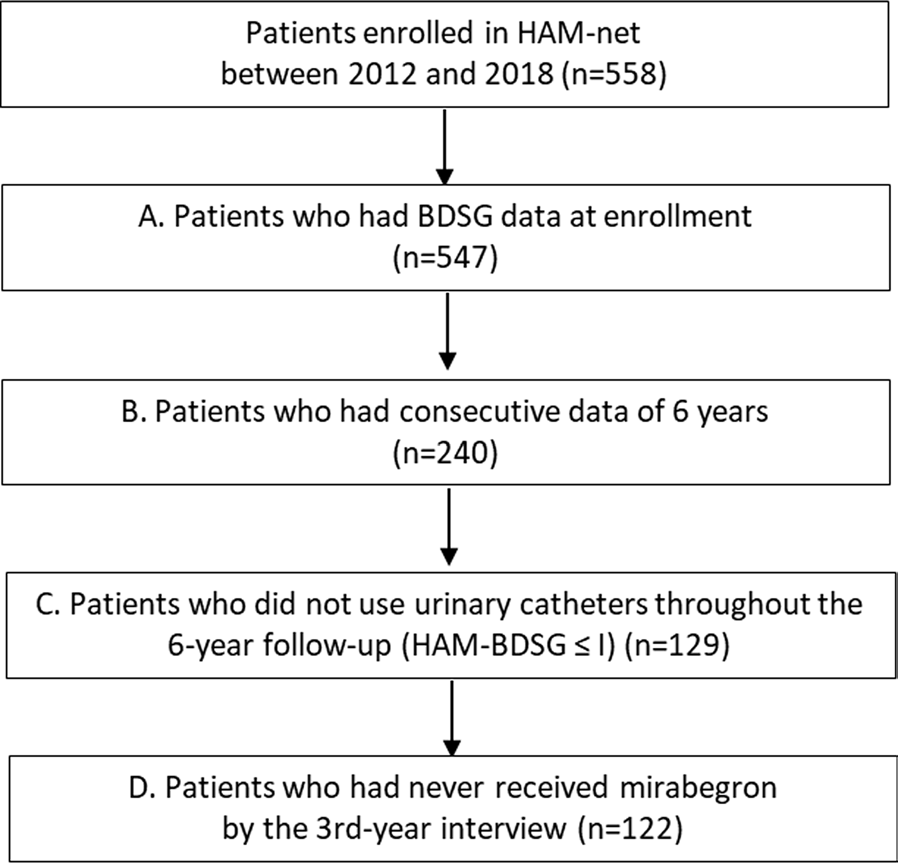
Study flow chart. HAM-BDSG, HAM/TSP-bladder dysfunction severity grade

Of the 558 patients enrolled in HAM-net between April 1, 2012, and December 31, 2018, 547 patients had HAM-BDSG data at enrollment (Set A of Fig. 1). We extracted information of patients who had responded consecutively to annual interviews over six years (Set B). Specific patient information was extracted from Set B for further analyses: those who did not use urinary catheters throughout the 6-year follow-up (Set C) and those who had never received mirabegron, a β3-adrenergic agonist for overactive bladder symptoms, by the 3rd-year interview (Set D). The St. Marianna University School of Medicine Bioethics Committee approved this study (Approval ID No. 2044), and all participants provided written informed consent.

### Valuables extracted from HAM-net

Demographic information, including age and sex, was extracted. To evaluate lower limb motor function, we extracted Osame Motor Disability Score (OMDS; Additional file 1: Table S2) [19], a scale specific to HAM/TSP that ranges from 0 to 13, with higher scores indicating greater walking disability. To assess urinary dysfunction and symptoms, we extracted information regarding the use of urinary catheters and medications for urinary dysfunction and scores of the following internationally validated urinary symptom measures: I-PSS [13], OABSS [14], and International Consultation on Incontinence Questionnaire-Short Form (ICIQ-SF) [21]. Missing data for urinary symptom scores were 1.7%, and those for other valuables were < 1.0%; missing data were not imputed.

### Bladder dysfunction symptom score and severity grade for HAM/TSP

We assessed urinary dysfunction severity using the HAM-BDSS (Additional file 1: Table S1) and HAM-BDSG (Additional file 1: Figure S1) [18]. These measures were recently developed to assess urinary dysfunction in patients with HAM/TSP. HAM-BDSS is composed of eight items derived from I-PSS and OABSS, with scores ranging from 0 to 40 points, where higher scores indicate greater symptom severity. Thus, HAM-BDSS can be calculated from I-PSS and OABSS data. The questions of the HAM-BDSS are subdivided into those related to storage and voiding symptoms (four questions each).

HAM-BDSG comprises four grades (grades 0–III) based on the presence or absence of urinary symptoms or medications and dependency on urinary catheters.

- Grade III: patients who use indwelling catheters.
- Grade II: patients who require intermittent catheters (where grade IIa patients have urine release control and grade IIb patients lack urine release control).
- Grade I: patients who do not use urinary catheters but have urinary symptoms or take medications.
- Grade 0: patients who do not use urinary catheters, have no urinary complaints, and take no medications for urinary symptoms.

Of note, HAM-BDSS of patients with HAM-BDSG 0 is not always zero because judging the presence or absence of urinary symptoms depends on patients’ sense and recognition.

### Outcomes

The outcomes of interest were: 1) prevalence and characteristics of urinary dysfunction in patients with HAM/TSP; 2) associations between urinary and gait dysfunction severity; 3) urinary dysfunction progression; and 4) treatment effects on urinary symptoms.

### Statistical analysis

Patient characteristics were summarized using means and standard deviations (SD) or medians with interquartile ranges (IQR) for continuous variables and proportions for categorical variables. To assess the trends of OMDS and HAM-BDSG, we divided OMDS into four levels (≤ 4, 5, 6, ≥ 7) and analyzed the association with HAM-BDSG using the Mantel–Haenszel test for trend. We evaluated the association between OMDS (≤ 4, 5, 6, ≥ 7) and HAM-BDSS using the Jonckheere-Terpstra trend test. Changes in HAM-BDSS in each participant were evaluated using paired t-test and repeated measures analysis of variance. We retrospectively evaluated associations between improvement in the fourth-year HAM-BDSS and mirabegron administration using multivariable linear regression models, adjusted by the third-year HAM-BDSS. The influence of potential confounders was analyzed using incremental adjustments: model 1 included third-year HAM-BDSS and initiation of mirabegron in the fourth year; model 2 was model 1 plus adjustments for age and sex; and model 3 was model 2 plus adjustments for other medications for urinary symptoms (i.e., α1 adrenergic-receptor antagonists, anticholinergics, and cholinergic agonists). We performed statistical analyses using IBM SPSS statistics version 25 and R version 3.4.2. All *p*-values were two-sided, and a p-value lower than 0.05 was considered statistically significant.

## Results

### Patient characteristics

Table 1 summarizes the 547 patient characteristics (Set A). At enrollment, 503 patients (92.0%) had urinary symptoms or were taking medications (HAM-BDSG ≥ I). Mean (SD) age was 61.9 (10.7) years, and 74.6% of patients were female. The mean age of disease onset was 45.3 (14.8) years, higher in patients with HAM-BDSG III than patients with other grades. Mean disease duration was 16.6 (11.6) years, which increased with HAM-BDSG up to grade II; however, that of patients with HAM-BDSG III was shorter than that of patients with HAM-BDSG II. Similar to a previous report [18], HAM-BDSS of patients with HAM-BDSG II was lower than that of patients with HAM-BDSG I (median [IQR], 8.5 [2–17.8] vs. 19.0 [13.0–26.0]), which suggested that urinary catheterization improved urinary symptoms. Medications for urinary symptoms were administered to 34.0% of patients, of which α1 adrenergic agonists (12.4%) and anticholinergics (11.3%) were the most frequently prescribed medications.

**Table 1:** Patient characteristics at HAM-net enrollment

Characteristic	All (n = 547)	BDSG 0 (n = 44)	BDSG I (n = 358)	BDSG II (n = 127)	BDSG III (n = 18)
Age at enrollment (years), mean (SD)	61.9 (10.7)	57.5 (13.9)	61.7 (10.4)	63.2 (9.9)	68.5 (9.6)
Age at disease onset (years), mean (SD)	45.3 (14.8)	46.3 (15.0)	45.2 (14.1)	44.0 (16.0)	53.4 (15.7)
Disease duration (years), mean (SD)	16.6 (11.6)	11.1 (8.5)	16.4 (11.2)	19.2 (12.7)	14.3 (11.7)
Female sex, n (%)	408 (74.6%)	31 (70.5%)	259 (72.3%)	103 (81.1%)	15 (83.3%)
OMDS, median (IQR)	5.0 (4–6)	4.0 (2–5)	5.0 (4–6)	6.0 (5–8)	10.0 (6–12)
Urinary symptom score					
HAM-BDSS					
Total score, mean (SD)	16.0 (10.1)	6.9 (6.6)	19.1 (9.1)	10.3 (9.6)	–
median (IQR)	16.0 (7–24)	7.0 (1–9.8)	19.0 (13–26)	8.5 (2–17.8)	–
Storage symptom score, mean (SD)	7.6 (5.3)	3.3 (2.8)	8.7 (5.1)	6.0 (5.4)	–
Median (IQR)	7.0 (3–12)	3.0 (1–5)	9.0 (5–13)	4.0 (1.3–10)	–
Voiding symptom score, mean (SD)	8.0 (6.7)	3.6 (4.9)	10.3 (6.2)	4.3 (6.0)	–
Median (IQR)	7.0 (1–15)	1.0 (0–5.8)	10.0 (5–15)	0 (0–8)	–
I-PSS, mean (SD)	13.5 (9.1)	6.1 (5.7)	16.5 (8.2)	8.0 (8.4)	–
Median (IQR)	13.0 (5–21)	6.0 (1–8)	17.0 (10–23)	4.0 (1–15)	–
OABSS, mean (SD)	6.0 (4.1)	2.7 (2.5)	6.7 (4.0)	5.0 (4.2)	–
Median (IQR)	6.0 (2–9)	2.5 (1–4)	7.0 (3–10)	4.0 (2–8)	–
ICIQ-SF, mean (SD)	6.0 (6.1)	2.3 (4.3)	6.6 (6.0)	5.7 (6.3)	6.5 (7.5)
Median (IQR)	5.0 (0–11)	0 (0–3)	7.0 (0–11)	3.5 (0–11)	6.0 (0–13.5)
Steroid therapy, n (%)	235 (43.0%)	13 (29.5%)	167 (46.6%)	48 (37.8%)	7 (38.9%)
Medication for urinary dysfunction, n (%)	186 (34.0%)	0 (0.0%)	145 (40.5%)	38 (29.9%)	3 (16.7%)
α1 adrenergic-receptor antagonists, n (%)	68 (12.4%)	0 (0.0%)	60 (16.8%)	8 (6.3%)	0 (0.0%)
β3-adrenoceptor agonists, n (%)	31 (5.7%)	0 (0.0%)	27 (7.5%)	4 (3.1%)	0 (0.0%)
Anticholinergics, n (%)	62 (11.3%)	0 (0.0%)	43 (12.0%)	19 (15.0%)	0 (0.0%)
Cholinergic agonists, n (%)	27 (4.9%)	0 (0.0%)	23 (6.4%)	4 (3.1%)	0 (0.0%)
Others, n (%)	34 (6.2%)	0 (0.0%)	22 (6.1%)	9 (7.1%)	3 (16.7%)

Values are expressed as mean (standard deviation), median (interquartile range), or number (proportion)

HAM-BDSG, HAM/TSP-bladder dysfunction severity grade; HAM-BDSS, HAM/TSP-bladder dysfunction symptom score

### Associations between urinary and gait dysfunction severity

**Fig. 2:**
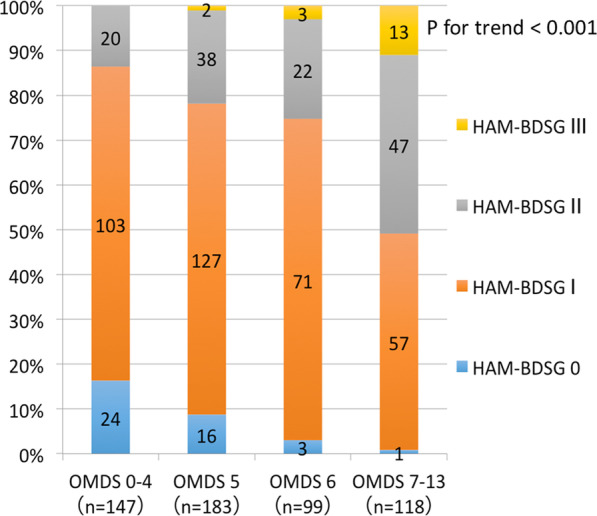
Association between OMDS and HAM-BDSG. Numbers and proportions of patients in each HAM-BDSG are shown according to the OMDS categories of 0–4, 5, 6, and 7–13 (n = 547). The trend of OMDS and HAM-BDSG was analyzed using the Mantel–Haenszel test for trend. HAM-BDSG, HAM/TSP-bladder dysfunction severity grade; OMDS, Osame Motor Disability Score

**Fig. 3:**
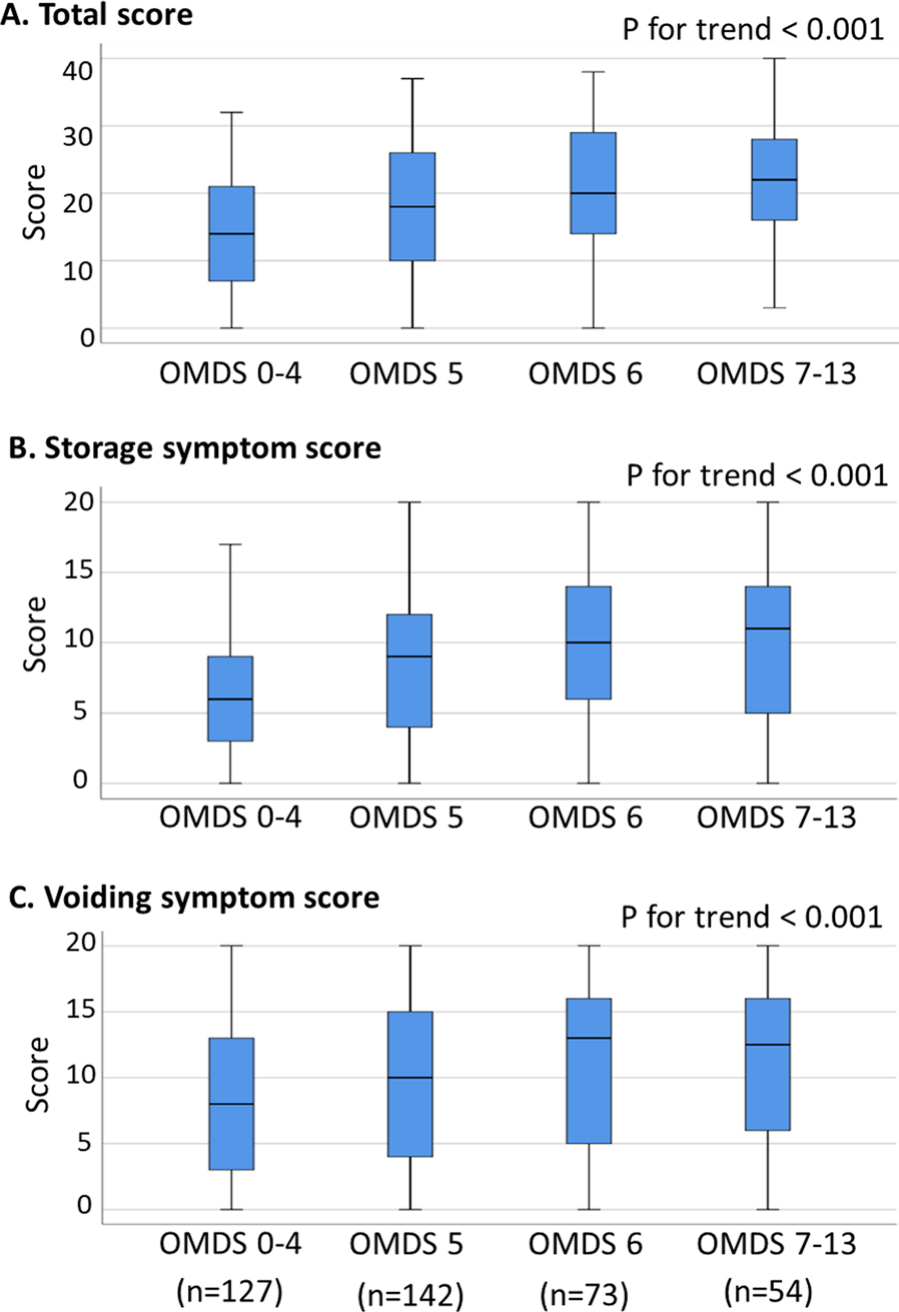
Associations between OMDS and HAMBDSS. **A**–**C** Distributions of HAM-BDSS total and subscores are summarized according to the OMDS categories of 0–4, 5, 6, and 7–13 (n = 396). Median, interquartile, maximum, and minimum values are shown. The association between OMDS and HAM-BDSS was evaluated using the Jonckheere-Terpstra trend test. HAM-BDSS, HAM/TSP-bladder dysfunction symptom score; OMDS, Osame Motor Disability Score

First, we evaluated the association between OMDS and HAM-BDSG using enrollment data (Set A). The proportion of patients with higher HAM-BDSG increased significantly with worsening OMDS (*p* < 0.001; Fig. 2). Next, we assessed the association between OMDS and HAM-BDSS in patients with HAM-BDSG ≤ I (i.e., those not using urinary catheters). HAM-BDSS and sub-scores were also worse in patients with higher OMDS (*p* < 0.001; Fig. 3). Collectively, urinary dysfunction in HAM/TSP tended to progress with worsening gait function. However, results also showed that urinary symptom scores were widely distributed even among patients with similar motor dysfunction levels.

### Progression of urinary dysfunction over six years

**Fig. 4.**
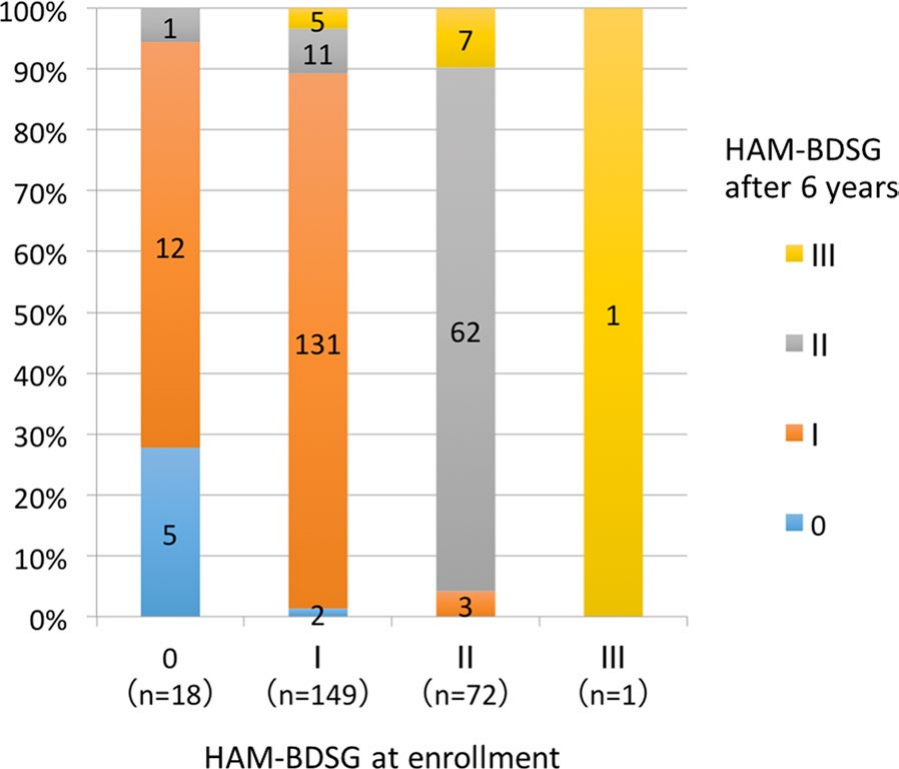
6-year change in HAM-BDSG. Numbers and proportions of patients with each HAM-BDSG 6 years after enrollment are shown according to HAM-BDSG at enrollment (n = 240). HAM-BDSG, HAM/TSP-bladder dysfunction severity grade

We assessed the change in HAM-BDSG from enrollment to the sixth year using data of those followed for six years (Set B). Although there was only one patient with HAM-BDSG III, baseline characteristics of patients with HAM-BDSG 0–II (Additional file 1: Table S3) were similar to those of Set A. Among the 18 patients with HAM-BDSG 0 at enrollment, 12 patients (66.7%) developed new urinary symptoms (HAM-BDSG I), and one patient (5.6%) initiated intermittent catheterization (HAM-BDSG II; Fig. 4). Of the 149 patients with HAM-BDSG I, 11 (7.4%) and five (3.4%) patients became dependent on intermittent and indwelling catheters (HAM-BDSG III), respectively. Seven patients (9.7%) with HAM-BDSG II progressed to HAM-BDSG III.

### Treatment effect of urinary catheters

**Fig. 5:**
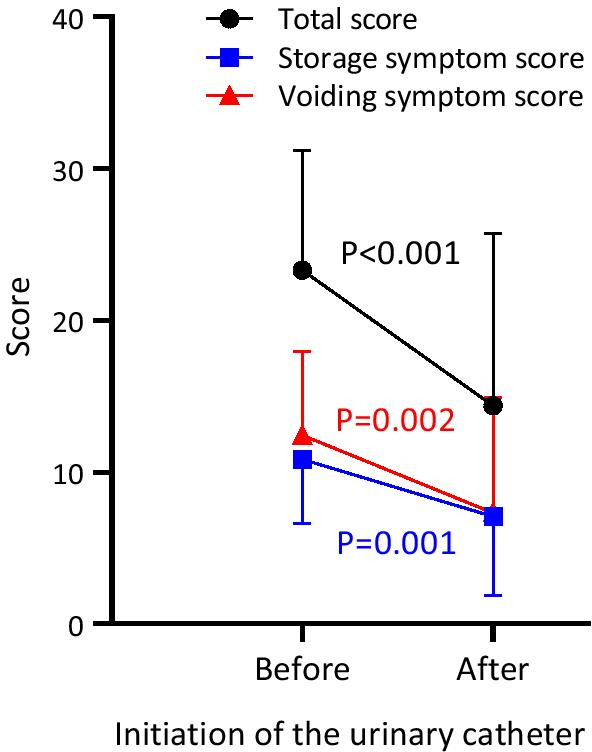
Improvement in HAM-BDSS by urinary catheterization. Mean (SD) HAM-BDSS for years before and after initiating urinary catheterization (n = 27). Changes in HAM-BDSS were evaluated using paired t-test. HAM-BDSS, HAM/TSP-bladder dysfunction symptom score

Because enrollment data suggested that intermittent catheters effectively relieve urinary symptoms, we examined changes in HAM-BDSS in those who initiated catheterization during follow-up. Total, storage, and voiding symptom scores significantly improved after initiating catheterization (mean [SD] change in total score: − 8.93 [10.78], *p* < 0.001; storage: − 3.78 [4.98], *p* = 0.001; voiding: − 5.15 [7.76], *p* = 0.002; Fig. 5).

### Changes in HAM-BDSS and the therapeutic effect of mirabegron

**Fig. 6.**
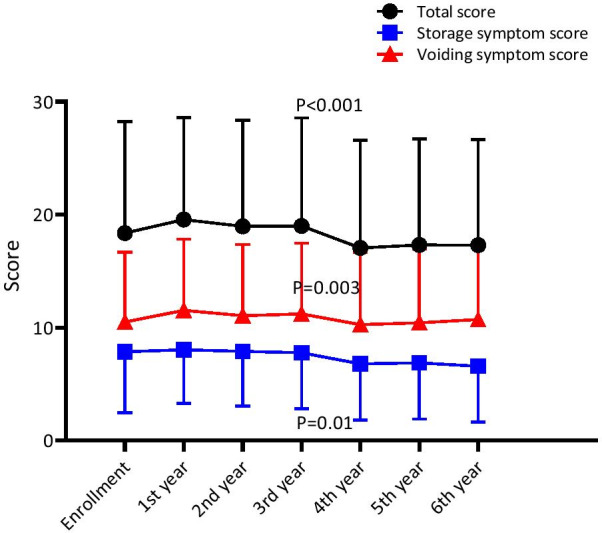
6-year change in HAM-BDSS in patients with HAM-BDSG 0–I. Mean (SD) HAM-BDSS in patients who remained at HAM-BDSG 0 or I throughout the 6-year follow-up (n = 129). During follow-up, changes in HAM-BDSS were statistically significant (repeated measures analysis of variance: *p* < 0.001 for total and storage symptom scores; *p* = 0.008 for voiding symptom score). P values in the graph reflect the statistical significance of changes from the third to the fourth year analyzed using paired t-test. HAM-BDSG, HAM/TSP-bladder dysfunction severity grade; HAM-BDSS, HAM/TSP-bladder dysfunction symptom score

**Fig. 7:**
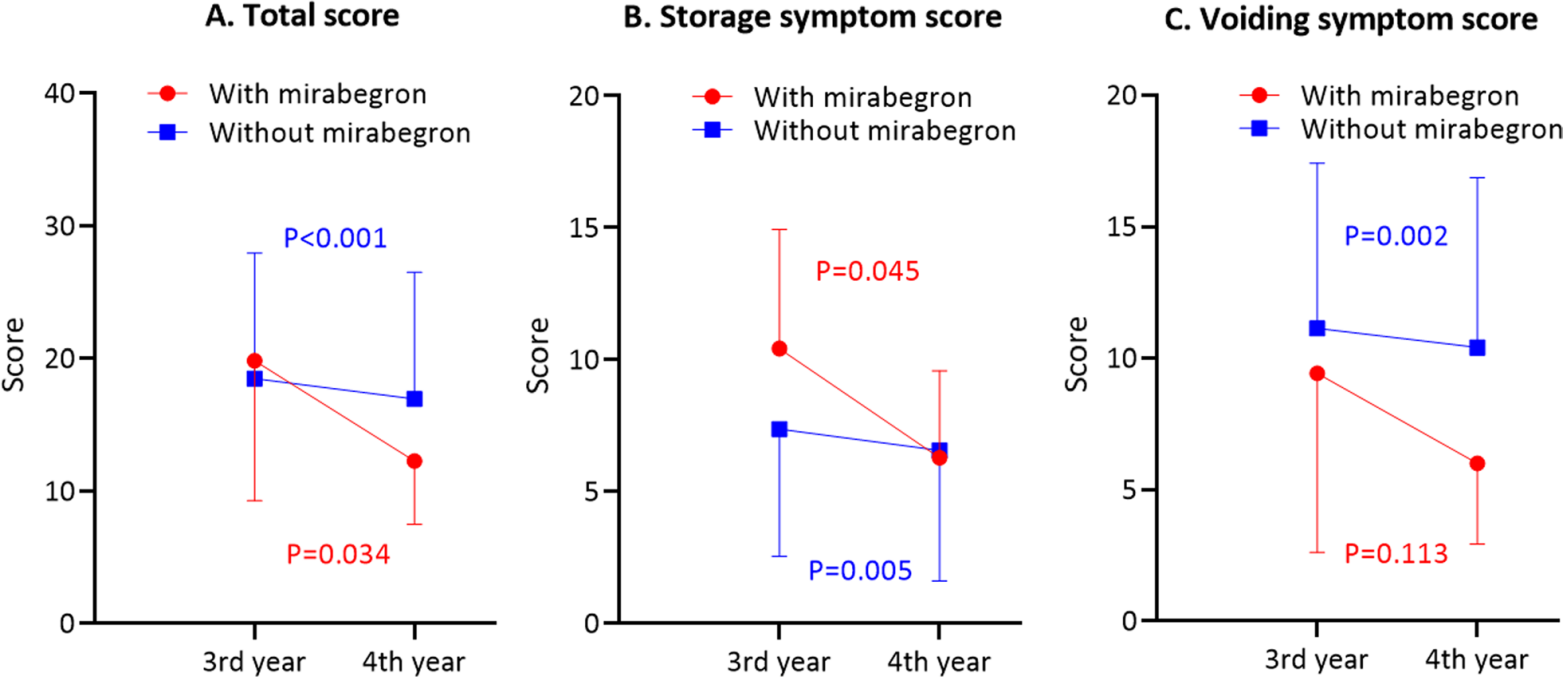
Changes in HAM-BDSS by mirabegron. **A**-**C** Mean (SD) HAM-BDSS total and subscores at the third- and fourth-year interviews in patients who did and did not initiate mirabegron in the fourth year (with mirabegron, n = 7; without mirabegron, n = 115). Changes within groups were evaluated using paired t-test. HAM-BDSS, HAM/TSP-bladder dysfunction symptom score

Because urinary catheterization significantly influenced HAM-BDSS, we assessed the 6-year changes in HAM-BDSS in only those who remained at HAM-BDSG 0 or I (Set C). Although HAM-BDSS was stable or slightly worsened up to the third year, the score significantly improved in the fourth year (Fig. 6). We speculated that changes in medication might have influenced HAM-BDSS and found that mirabegron was newly prescribed to seven patients in the fourth year. In contrast, other medications were largely unchanged (Additional file 1: Table S4). Therefore, we extracted information on 122 patients who had not received mirabegron by the third year to evaluate mirabegron’s effect on the fourth-year HAM-BDSS (Set D). Patient characteristics were similar between those with and without a new prescription of mirabegron (Additional file 1: Table S5). The fourth-year HAM-BDSS significantly improved in patients prescribed mirabegron, although change in voiding symptom score was not significant (mean [SD] change of the total score: − 7.57 [7.30], *p* = 0.034; storage: − 4.14 [4.34], *p* = 0.045; voiding: − 3.43 [4.89], *p* = 0.113; Fig. 7). Changes in HAM-BDSS in those not taking mirabegron were also significant, presumably because of the large sample size. However, these changes were small and were not considered clinically meaningful (mean [SD] change of the total score: − 1.55 [4.20], *p* < 0.001; storage: − 0.82 [3.04], *p* = 0.005; voiding: − 0.73 [2.42], *p* = 0.002). Finally, we performed multivariable linear regression to analyze the effect of mirabegron, adjusted by the third-year HAM-BDSS (Table 2). Results showed that initiation of mirabegron was significantly associated with better scores for all three measures, even after adjustment of the potential confounders of age, sex, and other medications for urinary symptoms (coefficient [95% confidence interval] of the total score: − 5.82 [− 9.13 to − 2.51], *p* = 0.001; storage: − 2.72 [− 5.02 to − 0.42], *p* = 0.021; voiding: − 2.81 [− 4.80 to − 0.82], *p* = 0.006).

**Table 2:** Multivariable linear regression analysis to evaluate the therapeutic effect of mirabegron on the fourth-year HAM-BDSS

Variable	Model 1			Model 2			Model 3		
	β	95%CI	p value	β	95%CI	p value	β	95%CI	p value
**Total score**									
Mirabegron started in 4th year	− 5.86	− 9.15, − 2.57	0.001	− 5.79	− 9.10, − 2.49	0.001	− 5.82	− 9.13, − 2.50	0.001
HAM-BDSS (3rd year)	0.88	0.80, 0.96	< 0.001	0.87	0.79, 0.95	< 0.001	0.86	0.78, 0.95	< 0.001
Age (1-year increase)	–	–	–	− 0.02	− 0.09, 0.05	0.592	− 0.02	− 0.08, 0.05	0.673
Sex (female vs. male)	–	–	–	− 0.93	− 2.61, 0.77	0.281	− 0.79	− 2.54, 0.96	0.373
α1 adrenergic-receptor antagonists	–	–	–	–	–	–	1.80	− 0.45, 4.03	0.115
Cholinergic agonists	–	–	–	–	–	–	− 1.75	− 5.44, 1.94	0.350
Anticholinergics	–	–	–	–	–	–	0.37	− 1.97, 2.71	0.752
**Storage symptom score**									
Mirabegron started in 4th year	− 2.73	− 5.06, − 0.41	0.022	− 2.64	− 4.97, − 0.32	0.026	− 2.72	− 5.02, − 0.42	0.021
HAM-BDSS (3rd year)	0.81	0.69, 0.92	< 0.001	0.79	0.67, 0.90	< 0.001	0.79	0.68, 0.91	< 0.001
Age (1-year increase)	–	–	–	–0.002	− 0.05, 0.05	0.926	0.00	− 0.05, 0.05	0.994
Sex (female vs. male)	–	–	–	− 0.94	− 2.11, 0.23	0.115	− 1.04	− 2.24, 0.16	0.088
α1 adrenergic-receptor antagonists	–	–	–	–	–	–	0.85	− 0.68, 2.38	0.275
Cholinergic agonists	–	–	–	–	–	–	− 1.45	− 4.00, 1.09	0.261
Anticholinergics	–	–	–	–	–	–	1.44	− 0.17, 3.05	0.079
**Voiding symptom score**									
Mirabegron started in 4th year	− 2.83	− 4.81, − 0.87	0.005	− 2.83	− 4.82, − 0.84	0.006	− 2.81	− 4.80, − 0.82	0.006
HAM-BDSS (3rd year)	0.92	0.85, 0.99	< 0.001	0.92	0.84, 0.99	< 0.001	0.91	0.83, 0.99	< 0.001
Age (1-year increase)	–	–	–	− 0.01	− 0.05, 0.03	0.626	− 0.01	− 0.05, 0.03	0.662
Sex (female vs male)	–	–	–	− 0.02	− 1.04, 0.99	0.963	0.22	− 0.83, 1.26	0.679
α1 adrenergic-receptor antagonists	–	–	–	–	–	−	0.82	− 0.53, 2.17	0.232
Cholinergic agonists	–	–	–	–	–	–	− 0.15	− 2.37, 2.07	0.893
Anticholinergics	–	–	–	–	–	–	− 1.06	− 2.46, 0.35	0.139

Multivariable linear regression analysis adjusted by the third-year HAM-BDSS: model 1 included the third-year HAM-BDSS and initiation of mirabegron in the fourth year; model 2 was model 1 plus adjustments for age and sex; model 3 was model 2 plus an adjustment for other medications for urinary symptoms

HAM-BDSS, HAM/TSP-bladder dysfunction symptom score

## Discussion

In this study, we characterized the urinary dysfunction of patients with HAM/TSP using comprehensive assessment measures that have recently been developed specifically for this patient population. As many as 92% of patients had urinary symptoms an average of 16.6 years after disease onset. This high incidence is similar to previous reports [7, 22].

Although disease duration increased with worsening HAM-BDSG up to grade II, it was shorter in patients with HAM-BDSG III than those with HAM-BDSG II, indicating there was a subset of patients who experienced rapid progression of urinary dysfunction. Moreover, symptom onset in patients with HAM-BDSG III was later than that in patients with other grades. Therefore, a later onset of HAM/TSP may be a risk factor for rapid urinary dysfunction progression, which has been reported previously for gait dysfunction [9, 23]. However, these findings should be interpreted with caution: disease duration, which was calculated from age at disease onset and at enrollment in HAM-net, has risk of recall bias; furthermore, older age may increase the use of indwelling catheters independent of HAM/TSP (e.g. concomitant urinary diseases, such as benign prostatic hypertrophy, and failure to learn intermittent catheterization).

We also evaluated the association between gait and urinary dysfunction severity. Although no significant association was found in a previous report, likely due to the small sample size (n = 60) [11], we demonstrated a robust correlation between the severity of gait and urinary dysfunction (Figs. 2 and 3). Urinary dysfunction in our patients tended to progress with worsening gait function. However, our data also showed that the degree of urinary symptom severity varied substantially across patients, even in those with similar motor dysfunction; moreover, some patients had severe urinary symptoms despite having mild gait dysfunction. Therefore, a comprehensive assessment of urinary symptoms is crucial, regardless of motor dysfunction severity.

We characterized urinary dysfunction progression using prospectively collected data and found that 66.7% of patients without urinary symptoms developed new symptoms within six years. Furthermore, 7.4% and 3.4% of those with urinary symptoms became dependent on intermittent and indwelling catheters, respectively (Fig. 4). In a retrospective study by Mori et al., approximately 70% of patients required urinary catheters long-term [9], which was likely attributable to the study population characteristics and longer follow-up period. Moreover, the study might have included more severe patients because most patients had undergone a urodynamic study in the urology department. Our study may more accurately reflect the clinical course of patients with HAM/TSP because our data were prospectively collected from a larger cohort of patients with a wider range in severity.

Intermittent catheterization is the cornerstone of management for severe voiding dysfunction [24, 25], and timely initiation is important because voiding dysfunction can lead to serious complications, such as kidney dysfunction and urinary tract infection. However, patient effort and understanding are necessary to start catheterization because the treatment is invasive and requires training. We quantitatively demonstrated that catheterization significantly relieves voiding and storage symptoms (Fig. 5). Visualizing the benefits of catheterization may help encourage patients to start catheterization treatment.

It is difficult to conduct randomized controlled trials for rare diseases, and there have not been any prospective comparative studies on treatments for neurogenic bladder in patients with HAM/TSP. Matsuo et al. recently conducted a single-arm 12-week interventional study and reported that mirabegron effectively relieved overactive bladder symptoms in patients with HAM/TSP [26]. In the present study, we retrospectively evaluated mirabegron’s therapeutic efficacy because patients receiving the medication increased during follow-up. HAM-BDSS total and storage symptom scores significantly improved after initiation of mirabegron (Fig. 7). Moreover, mirabegron was effective, even after adjusting for potential confounders (Table 2). Although our study was retrospective, we included patients without mirabegron in the analysis as a comparator. In the regression analysis, the effect of mirabegron on voiding symptom score was also significant, which did not occur in a previous study [26]. The reason for this is unclear, although the positive effect of mirabegron on storage symptoms, such as the reduction in urination frequency, may have indirectly influenced patients’ subjective assessment of voiding symptoms. Further studies in larger sample sizes are necessary to confirm the efficacy.

Our study has several limitations. As we included only Japanese people, the generalizability of our findings may be limited. Furthermore, we did not have urodynamic study data. Urinary symptoms are common, even in the general population and asymptomatic HTLV-1 carriers [10, 22, 27]. Therefore, we could not conclude that neurogenic bladder caused urinary symptoms in our patients. Because the efficacy analysis of mirabegron included only seven patients, the reliability of the results is limited. Nevertheless, despite these shortcomings, to the best of our knowledge, this is the largest study on urinary dysfunction in patients with HAM/TSP, which included more than 500 patients throughout Japan. Thus, our cohort is a representative population of patients with HAM/TSP. Moreover, most previous studies have been cross-sectional or retrospective, whereas we prospectively uniformly collected patient data over six years. We revealed various aspects of urinary dysfunction in these patients and the importance of observational studies using real-world data for rare diseases.

## Conclusions

We described the characteristics of urinary dysfunction in patients with HAM/TSP, which included prevalence, longitudinal changes, associations with gait dysfunction, and treatment efficacy. Urinary dysfunction affected most patients, and its severity varied substantially. Therefore, a comprehensive assessment is required for accurate evaluation and effective management. We demonstrated that effective utilization of real-world data is key to establishing valuable evidence for rare diseases, such as HAM/TSP.

## Supplementary Information

**Additional file 1. Supplementary methods**. HAM-net.** Table S1**. HAM/TSP-bladder dysfunction symptom score (HAM-BDSS).** Table S2**. Osame Motor Disability Score (OMDS).** Table S3**. Baseline characteristics of patients with the consecutive 6-year follow-up (Set B).** Table S4**. Medications for urinary symptoms prescribed in patients with HAMBDSG 0–I (Set C).** Table S5**. Characteristics of patients for the analysis of the therapeutic effects of mirabegron (Set D).** Figure S1**. HAM/TSP-bladder dysfunction severity grade (HAM-BDSG).

## Data Availability

The datasets analyzed during the current study are available from the corresponding author on reasonable request.

## Abbreviations

HAM-BDSG: HAM-bladder dysfunction severity grade
HAM-BDSS: HAM/TSP-bladder dysfunction symptom score
HAM/TSP: HTLV-1-associated myelopathy/tropical spastic paraparesis
HTLV-1: Human T-cell leukemia virus type 1
ICIQ-SF: International Consultation on Incontinence Questionnaire-Short Form
I-PSS: International Prostate Symptom Score
NBSS: Neurogenic Bladder Symptom Score
OABSS: Overactive Bladder Symptom Score
OMDS: Osame Motor Disability Score
