# Supplementary material for: Clinical course of neurogenic bladder dysfunction in human T-cell leukemia virus type-1-associated myelopathy/tropical spastic paraparesis: a nationwide registry study in Japan
Source: Orphanet J Rare Dis. 2021 Aug 9;16:355. doi: 10.1186/s13023-021-01990-3 (PMC8351405; doi:10.1186/s13023-021-01990-3)
Supplement: Supplementary file 1 — Additional file 1. Supplementary methods. HAM-net. Table S1. HAM/TSP-bladder dysfunction symptom score (HAM-BDSS). Table S2. Osame Motor Disability Score (OMDS). Table S3. Baseline characteristics of patients with the consecutive 6-year follow-up (Set B). Table S4. Medications for urinary symptoms prescribed in patients with HAMBDSG 0–I (Set C). Table S5. Characteristics of patients for the analysis of the therapeutic effects of mirabegron (Set D). Figure S1. HAM/TSP-bladder dysfunction severity grade (HAM-BDSG). [file 13023_2021_1990_MOESM1_ESM.docx]

**Supplementary information**

**Additional file 1**

**Supplementary methods**

HAM-net

**Supplementary tables**

Table S1. HAM/TSP-bladder dysfunction symptom score (HAM-BDSS)

Table S2. Osame Motor Disability Score (OMDS)

Table S3. Baseline characteristics of patients with the consecutive 6-year follow-up (Set B)

Table S4. Medications for urinary symptoms prescribed in patients with HAM-BDSG 0–I (Set C)

Table S5. Characteristics of patients for the analysis of the therapeutic effects of mirabegron (Set D)

**Supplementary figure**

Figure S1. HAM/TSP-bladder dysfunction severity grade (HAM-BDSG)

**Supplementary methods**

**HAM-net**

HAM-net is a nationwide registration system for patients with HAM/TSP that was introduced at St. Marianna University in Japan in March 2012. The number of registered patients (558 patients in 2019) is estimated to be one-fifth or one-sixth of the total patient population in Japan [1]. Patients are recruited throughout Japan from informational leaflets distributed to patients at clinics, patient meetings, and board-certified neurologists. Patients with HAM/TSP can apply to the HAM-net registration center via telephone, fax, or e-mail. Trained nurses and coordinators conduct annual telephone interviews with patients in a uniform manner. Data collected include age, sex, disease duration, and past and present medical conditions, such as symptoms, comorbidities, and medications. Motor disability is evaluated using the Osame Motor Disability Score (OMDS). Urinary symptoms are assessed using Japanese versions of internationally validated urinary scales validated for the general native Japanese-speaking population, including the International Prostate Symptom Score (I-PSS), the Overactive Bladder Symptom Score (OABSS), and International Consultation on Incontinence Questionnaire-Short Form (ICIQ-SF).

**Reference**

1. Coler-Reilly ALG, Yagishita N, Suzuki H, Sato T, Araya N, Inoue E, et al. Nation-wide epidemiological study of Japanese patients with rare viral myelopathy using novel registration system (HAM-net). Orphanet J Rare Dis. 2016;11:69.

**Supplementary tables**

**Table S1. HAM/TSP-bladder dysfunction symptom score (HAM-BDSS)**

| **No** | **Symptom** | **Not at All** | **Less than 1 in 5 times** | **Less than half the time** | **About half the time** | **More than half the time** | **Almost always** |
| --- | --- | --- | --- | --- | --- | --- | --- |
| **1** | In the past month, how often have you had to urinate less than every 2 hours? | 0 | 1 | 2 | 3 | 4 | 5 |
|  |  | **None** | **1 time** | **2 times** | **3 times** | **4 times** | **5 or more times** |
| **2** | In the past month, how many times did you typically get up at night to urinate? | 0 | 1 | 2 | 3 | 4 | 5 |
|  |  | **Not at all** | **Less than once a week** | **Once a week or more** | **About once a day** | **2–4 times a day** | **5 times a day or more** |
| **3** | In the past week, how often do you have a sudden desire to urinate, which was difficult to defer? | 0 | 1 | 2 | 3 | 4 | 5 |
| **4** | In the past week, how often do you leak urine because you could not defer the sudden desire to urinate? | 0 | 1 | 2 | 3 | 4 | 5 |
| **Storage symptoms score** | | | | | | | **/20** |
| **No** | **Symptom** | **Not at all** | **Less than 1 in 5 times** | **Less than half the time** | **About half the time** | **More than half the time** | **Almost always** |
| **5** | In the past month, how often have you had the sensation of not completely emptying your bladder? | 0 | 1 | 2 | 3 | 4 | 5 |
| **6** | In the past month, when urinating, how often have you found yourself to have stopped and then resumed several times? | 0 | 1 | 2 | 3 | 4 | 5 |
| **7** | In the past month, how often have you had a weak urinary stream? | 0 | 1 | 2 | 3 | 4 | 5 |
| **8** | In the past month, how often have you had to strain to start urination? | 0 | 1 | 2 | 3 | 4 | 5 |
| **Voiding symptom score** | | | | | | | **/20** |
| **Total score** | | | | | | | **/40** |

Reprinted with permission from Yamakawa N, Yagishita N, Matsuo T, Yamauchi J, Ueno T, Inoue E, et al. Creation and validation of a bladder dysfunction symptom score for HTLV-1-associated myelopathy/tropical spastic paraparesis. Orphanet J Rare Dis. 2020;15:175.

**Table S2. Osame Motor Disability Score (OMDS)**

| **Grade** | **Motor disability** |
| --- | --- |
| 0 | No walking or running abnormalities |
| 1 | Normal gait but runs slowly |
| 2 | Abnormal gait (stumbling, stiffness) |
| 3 | Unable to run |
| 4 | Needs handrail to climb stairs |
| 5 | Needs a cane (unilateral support) to walk |
| 6 | Needs bilateral support to walk |
| 7 | Can walk 5–10 m with bilateral support |
| 8 | Can walk 1–5 m with bilateral support |
| 9 | Cannot walk, but able to crawl |
| 10 | Cannot crawl, but able to move using arms |
| 11 | Cannot move around, but able to turn over in bed |
| 12 | Cannot turn over in bed |
| 13 | Cannot even move toes |

**Table S3. Baseline characteristics of patients with the consecutive 6-year follow-up (Set B)**

| **Characteristic** | **All  (n = 240)** | **BDSG 0 (n = 18)** | **BDSG I (n = 149)** | **BDSG II (n = 72)** | **BDSG III (n = 1)** |
| --- | --- | --- | --- | --- | --- |
| **Age at enrollment (years), mean (SD)** | 61.1 (10.6) | 55.6 (15.3) | 61.3 (10.2) | 62.2 (10.0) | 54.0 |
| **Age at disease onset (years), mean (SD)** | 43.1 (14.9) | 42.8 (15.3) | 44.0 (14.2) | 41.2 (16.2) | 41.0 |
| **Disease duration (years), mean (SD)** | 18.1 (10.6) | 12.7 (8.4) | 17.3 (10.3) | 21.0 (11.1) | 13.0 |
| **Female sex, n(%)** | 183 (76.3%) | 12 (66.7%) | 109 (73.2%) | 61（84.7%） | 1（100） |
| **OMDS, median (IQR)** | 5.0 (5–6) | 4.5 (2–5) | 5.0 (5–6) | 6.0 (5–8) | 5.0 |
| **Urinary symptom score** |  |  |  |  |  |
| **HAM-BDSS** |  |  |  |  |  |
| **Total score, mean (SD)** | 17.0 (10.9) | 8.6 (7.8) | 21.1 (9.4) | 10.6 (10.4) | - |
| **median (IQR)** | 18.0 (7–26) | 9.0 (1–12) | 23.0 (14–28.5) | 7.5 (2–19.5) | - |
| **Storage symptom score, mean (SD)** | 8.2 (5.7) | 3.8 (3.6) | 9.5 (5.4) | 6.4 (5.9) | - |
| **median (IQR)** | 8.0 (3–13) | 3.0 (0.8–6.3) | 10.0 (5–13) | 5.0 (1–11) | - |
| **Voiding symptom score, mean (SD)** | 8.8 (6.9) | 4.8 (5.6) | 11.6 (5.8) | 4.2 (6.2) | - |
| **median (IQR)** | 10.0 (1–15) | 1.0 (0.8–10) | 13.0 (6.5–16) | 0 (0–7.5) | - |
| **I-PSS, mean (SD)** | 14.6 (9.8) | 7.5 (6.7) | 18.5 (8.3) | 8.3 (9.0) | - |
| **median (IQR)** | 15.0 (6–23) | 7.5 (1–12) | 20.0 (13–25) | 3.0 (1–16) | - |
| **OABSS, mean (SD)** | 6.4 (4.4) | 3.2 (3.1) | 7.2 (4.1) | 5.5 (4.6) | - |
| **median (IQR)** | 6.0 (2–10) | 3.0 (0.8–6) | 8.0 (3.5–11) | 4.0 (2–10) | - |
| **ICIQ-SF, mean (SD)** | 6.2 (6.2) | 1.7 (3.2) | 6.8 (6.0) | 6.1 (6.8) | 14.0 |
| **median (IQR)** | 5.5 (0–11) | 0 (0–3) | 7.0 (0–12) | 3.0 (0–12) | 14.0 |
| **Steroid therapy at registration, n(%)** | 102 (42.5%) | 8 (44.4%) | 67 (45.0%) | 26 (36.1%) | 1 (100%) |
| **Medication for urinary dysfunction at registration, n(%)** | 84 (35.0%) | 0 (0.0%) | 63 (42.3%) | 21 (29.2%) | 0 (0.0%) |
| **α1 adrenergic-receptor antagonists** | 29 (12.1%) | 0 (0.0%) | 27 (18.1%) | 2 (2.8%) | 0 (0.0%) |
| **β3-adrenoceptor agonists, n(%)** | 5 (2.1%) | 0 (0.0%) | 4 (2.7%) | 1 (1.4%) | 0 (0.0%) |
| **Anticholinergics, n(%)** | 29 (12.1%) | 0 (0.0%) | 18 (12.1%) | 11 (15.3%) | 0 (0.0%) |
| **Cholinergic agonists, n(%)** | 12 (5.0%) | 0 (0.0%) | 11 (7.4%) | 1 (1.4%) | 0 (0.0%) |
| **Others, n(%)** | 19 (7.9%) | 0 (0.0%) | 11 (7.4%) | 8 (11.1%) | 0 (0.0%) |

Values are expressed as mean (standard deviation [SD]), median (interquartile range [IQR]), and number (proportion).

HAM-BDSG, HAM/TSP-bladder dysfunction severity grade; HAM-BDSS, HAM/TSP-bladder dysfunction symptom score.

**Table S4. Medications for urinary symptoms prescribed to patients with HAM-BDSG 0–I (Set C)**

| **Drug classification** | **Generic name** | **Interview year** | | | | | | |
| --- | --- | --- | --- | --- | --- | --- | --- | --- |
|  |  | **Enrollment** | **1st** | **2nd** | **3rd** | **4th** | **5th** | **6th** |
| **α1-adrenergic-receptor　antagonists** | **Urapidil** | 11 | 12 | 13 | 14 | 17 | 20 | 23 |
|  | **Tamsulosin Hydrochloride** | 4 | 6 | 6 | 6 | 6 | 6 | 6 |
|  | **Silodosin** | 2 | 2 | 2 | 2 | 3 | 3 | 3 |
|  | **Prazosin Hydrochloride** | 2 | 4 | 3 | 3 | 3 | 4 | 4 |
|  | **Naftopidil** | 0 | 0 | 0 | 0 | 0 | 0 | 0 |
| **Cholinergic agonists** | **Distigmine Bromide** | 6 | 6 | 7 | 7 | 7 | 7 | 7 |
|  | **Bethanechol Chloride** | 2 | 3 | 3 | 3 | 3 | 3 | 3 |
| **Anticholinergics** | **Solifenacin Succinate** | 3 | 4 | 3 | 3 | 4 | 4 | 6 |
|  | **Imidafenacin** | 4 | 5 | 5 | 9 | 8 | 8 | 6 |
|  | **Propiverine Hydrochloride** | 2 | 3 | 2 | 2 | 2 | 2 | 2 |
|  | **Fesoterodine Fumarate** | 0 | 0 | 0 | 0 | 1 | 1 | 1 |
|  | **Oxybutynin hydrochloride** | 1 | 1 | 1 | 1 | 1 | 1 | 2 |
|  | **Tolterodine Tartrate** | 1 | 1 | 1 | 1 | 1 | 1 | 1 |
| **β3-adrenoceptor agonists** | **mirabegron** | **4** | **7** | **5** | **5** | **12** | **15** | **14** |

The columns show the number of patients who received each medication.

**Table S5. Characteristics of patients for the analysis of the therapeutic effect of mirabegron (Set D)**

| **Characteristic** | **Total (n = 122)** | **With mirabegron (n = 7)** | **Without mirabegron (n = 115)** |
| --- | --- | --- | --- |
| **Age (years), mean (SD)** | 66.2 (11.4) | 67.3 (5.3) | 66.2 (11.6) |
| **Age at disease onset (years), mean (SD)** | 44.2 (14.5) | 46.9 (13.5) | 44.0 (14.6) |
| **Disease duration (years), mean (SD)** | 22.1 (10.2) | 20.4 (10.4) | 22.2 (10.2) |
| **Female sex, n(%)** | 83 (68.0%) | 5 (71.4%) | 78 (67.8%) |
| **OMDS, median (IQR)** | 5.0 (4–6) | 5.0 (3–6) | 5.0 (4–6) |
| **HAM-BDSS** |  |  |  |
| **Total score, mean (SD)** | 18.6 (9.4) | 19.9 (10.6) | 18.5 (9.4) |
| **median (IQR)** | 19.0 (12–25) | 20.0 (8–33) | 19.0 (12–25) |
| **Storage symptom score, mean (SD)** | 7.6 (4.8) | 10.4 (4.5) | 7.4 (4.8) |
| **median (IQR)** | 7.5 (3.8–11) | 12.0 (6–13) | 7.0 (3–11) |
| **Voiding symptom score, mean (SD)** | 11.0 (6.3) | 9.4 (6.8) | 11.1 (6.3) |
| **median (IQR)** | 11.0 (5–16) | 7.0 (5–17) | 12.0 (5–16) |
| **α1 adrenergic-receptor antagonists, n(%)** | 23 (18.9%) | 1 (14.3%) | 22 (19.1%) |
| **Cholinergic agonists, n(%)** | 7 (5.7%) | 0 (0.0%) | 7 (6.1%) |
| **Anticholinergics, n(%)** | 16 (13.1%） | 1 (14.3%) | 15 (13.0%) |

Values are expressed as mean (SD), median (IQR), and number (proportion).

HAM-BDSS, HAM/TSP-bladder dysfunction symptom score.

**Supplementary figure**

**Figure S1. HAM/TSP-bladder dysfunction severity grade (HAM-BDSG)**


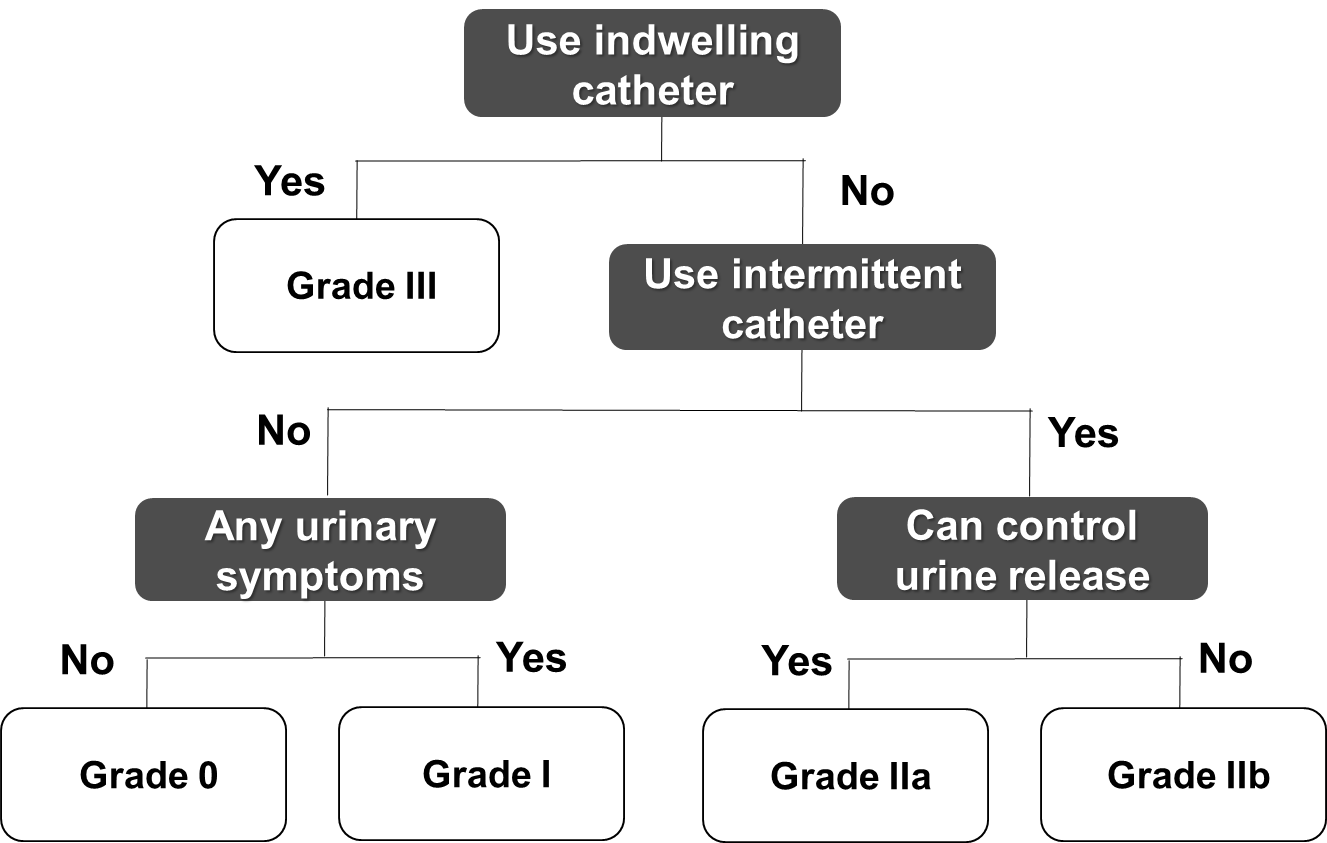


Patients who use indwelling catheters are classified as grade III, those who require intermittent catheters are classified as grade II (IIa for those who have urine release control and IIb for those who lack urine release control), those who do not use urinary catheters but have urinary symptoms or take medications are classified as grade I, and those who do not use urinary catheters, have urinary complaints, or take medications for urinary symptoms are classified as grade 0. Reprinted with permission from Yamakawa N, Yagishita N, Matsuo T, Yamauchi J, Ueno T, Inoue E, et al. Creation and validation of a bladder dysfunction symptom score for HTLV–1-associated myelopathy/tropical spastic paraparesis. Orphanet J Rare Dis. 2020;15:175.
